# Quantification of pathological gait parameter thresholds of idiopathic normal pressure hydrocephalus patients in clinical gait analysis

**DOI:** 10.1038/s41598-022-22692-1

**Published:** 2022-10-31

**Authors:** Ken Möhwald, Max Wuehr, Julian Decker, Eric-Manuel Asch, Fabian Schenkel, Ben Illigens, Roman Schniepp

**Affiliations:** 1 Department of Neurology, University Hospital, LMU Munich, Marchioninistr. 15, 81377 Munich, Germany; 2 German Center for Vertigo and Balance Disorders (DSGZ), University Hospital, LMU Munich, Marchioninistr. 15, 81377 Munich, Germany; 3 Division of Health Care Sciences, Center for Clinical Research and Management Education, Dresden International University, Dresden, Germany; 4 Schoen Klinik Bad Aibling, Kolbermoorer Str. 72, 83043 Bad Aibling, Germany; 5 Beth Israel Deaconess Medical Center, Harvard Medical School, 330 Brookline Avenue, Boston, MA 02215 USA

**Keywords:** Ageing, Geriatrics, Hydrocephalus, Movement disorders, Neurodegenerative diseases, Computational neuroscience

## Abstract

The aim of the study was to distinguish the hypokinetic gait disorder in idiopathic normal pressure hydrocephalus (NPH) patients from the gait decline in the elderly population by quantifying pathological gait parameter thresholds utilizing a multiple condition gait assessment. 55 NPH patients and 55 age-matched healthy subjects underwent a standardized gait assessment with eight gait conditions. Spatiotemporal gait parameters were assessed through a pressure-sensitive carpet. Statistical analysis consisted of a binary logistic regression (BLR) model, logistic curve-fit evaluated by a Chi-square goodness-of-fit-test, receiver operating characteristic models with area under the curves (AUC), and inverse BLR. Most discriminative gait parameter thresholds were observed in pace, gait cycle, and support gait domains. The most distinct gait conditions were preferred walking speed and semantic dual task. During preferred walking speed, the most significant gait parameter thresholds were stride length ≤ 1.02 m (sensitivity 0.93/specificity 0.91/AUC 0.96), gait velocity ≤ 0.83 m/s (0.80/0.91/0.93), double support phase ≥ 27.0% (0.96/0.76/0.91), and stride length coefficient of variation ≥ 3.4% (0.93/0.72/0.90). In conclusion, the hypokinetic gait disorder in NPH can be quantitatively differentiated from gait patterns of the elderly population. In future studies, this approach may be useful to differentiate clinical entities with similar gait disorders utilizing instrumented gait analysis procedures.

## Introduction

Idiopathic normal pressure hydrocephalus (NPH) is a chronic neurological disease characterized by gait disturbance, dementia, and urinary incontinence, which was first described by Adam and Hakim in the 1960s^1^. Impaired circulation of cerebrospinal fluid (CSF) and mostly normal to mildly increased cerebrospinal fluid pressure are considered to play a key role for the emergence of disease, thus, shunt surgery represents a potent and causative treatment option^2^.

Even though epidemiological data is sparse, NPH is a disease of the elderly population and is estimated to occur most frequently above the age of 60 years^3–5^. Although the overall prevalence appears to be low, the clinical routine often comprises an overestimation of suspected NPH. With the gait disorder being most prominent in NPH^3,6,7^, neurologists and neurosurgeons are challenged to identify and integrate gait features into differential diagnosis for NPH.

The gait disorder in NPH typically represents a hypokinetic gait pattern with slow gait velocity, decreased step length, increased double support phase, and stride width^8–11^. However, these features are ambiguous since they also occur as a result of the age-related decline in gait performance^12–14^. This may complicate clinical identification of NPH-related gait impairments, particularly during early stages of disease. Furthermore, a comprehensive and objectified quantification of gait performance in NPH is not only crucial for differential diagnosis, but also for the selection of eligible candidates for shunt placement and the monitoring of intervention effects after shunt surgery. There is an ongoing debate, whether the examination of gait performance in NPH during sensory disturbed or motor-cognitive dual tasks might increase the diagnostic power^15–17^. As common in patients with motor-cognitive gait disturbances, a decline of walking performance or the “stops walking while talking” phenomenon have been described in several studies^9,18^.

Therefore, we quantitatively characterized gait performance of patients with NPH and age-matched healthy controls during a comprehensive multiple condition gait assessment including walking with different speeds, sensory perturbations and walking with a second cognitive task (dual task). Based on this, the aim of this study was twofold:

1. To analytically evaluate the discriminative power of each collected gait feature with respect to a distinctive threshold with optimal balanced sensitivity and specificity for the identification of NPH in an elderly cohort.
2. To rank each feature with respect to its discriminative validity in order to inform clinicians about the most relevant gait parameters and examination conditions for this clinical decision setting.

## Methods

### Ethical approval and patient consent

All study procedures have been approved by the Ethics Committee of the University of Munich (reference number 34-16). The study was in accordance with the Declaration of Helsinki in its newest revision. Informed consent was obtained from all subjects and/or their legal guardian(s).

### Study cohort and data collection

One hundred ninety-four adult patients, who presented due to suspected NPH symptoms, were screened in our clinic. Fifty-five patient with NPH (mean age 72.6 ± 4.7 years, 18 females) with a mean duration of symptoms of 2.0 ± 1.5 years during the first clinical visit were included in the study according to previously published guidelines and criteria^19,20^. Exclusion criteria were secondary forms of hydrocephalus, other primary causes of gait impairment (e.g., comorbidities, disabilities or residual symptoms such as hemiparesis after stroke, etc.) and the inability to walk independently. Patients were followed up in the course of disease with evaluation of shunt candidacy (lumbar drainage, improvement in gait analysis and neuropsychological tests) and if they subsequently underwent a VP shunt surgery. For the control group, 55 age-matched healthy subjects (mean age 70.5 ± 7.6 years, 27 females) were recruited. Exclusion criteria were any morbidities with significant impact on locomotion.

### Gait analysis and parameters

Gait performance was investigated using a pressure-sensitive sensor carpet (6.7 m, GAITRite®, CIR Systems) and a concomitant 2D video recording. Patients and healthy subjects underwent a standardized gait protocol with eight different gait conditions: walking in preferred speed (PS), slow speed (SS), and maximum walking speed (MS); walking during head reclination (HR) and eyes closed (EC); walking during cognitive calculatory dual task (serial 7 subtractions; DTC), semantic dual task (verbal fluency; DTS), and motoric dual task (carrying a tray; DTM). For each condition, spatiotemporal gait parameters were calculated and analyzed. Parameters are summarized in five independent gait domains, which are based on a previous study^21^: (1) Pace: velocity (m/s), stride length (m), stride time (s). (2) Cycle: swing phase (%), double support phase (%). (3) Variability: stride length coefficient of variation (CV) (%), stride time CV (%), swing phase CV (%). (4) Asymmetry: stride length asymmetry (%), stride time asymmetry (%), swing phase asymmetry (%). (5) Support: stride width (m), stride width CV (%). Occurrences of “stops walking while talking” and freezing of gait phenomena were identified by two independent investigators (KM and RS) based on video recordings.

### Statistical analysis

**Figure 1:**
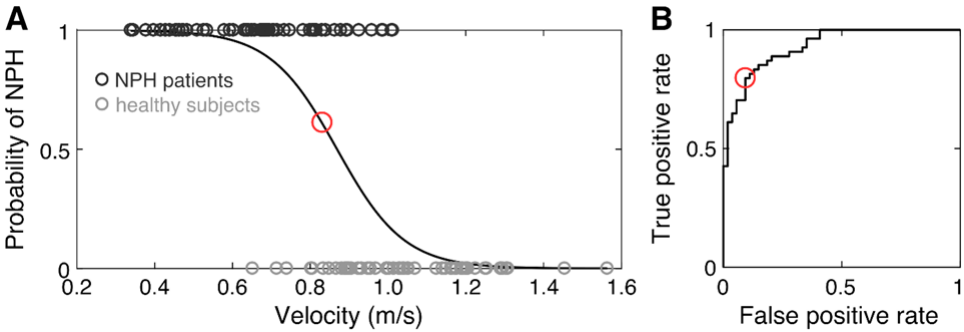
Illustration of the statistical methodology demonstrated on the example of gait velocity during preferred walking speed in m/s. Using a binary logistic regression (BLR) model, the distribution of the logistic curve-fit is depicted on the left-hand side (**A**) with 1 for NPH and 0 for being healthy. The corresponding receiver operating characteristic (ROC) curve is shown on the right-hand side (**B**). The determined gait parameter threshold (**A**) as well as the optimal operating point of the ROC curve (**B**) with balanced true positive rate (sensitivity) and false positive rate (1-specificity) are marked with a red circle.

Descriptive statistics are presented as mean ± standard deviation (SD). Statistical analysis was performed in correspondence to a previously proposed procedure^22^. For each collected gait feature a binary logistic regression model (BLR) was performed with respect to the binary outcome variable (0: healthy, 1: NPH). The validity of the logistic curve-fit was evaluated using a Chi-square goodness-of-fit test. Classification accuracy of the regression model was evaluated based on a receiver operating characteristic (ROC) procedure with respect to the area under the curve (AUC). The optimal operating point of the ROC curve with balanced sensitivity and specificity levels was determined. This point was then fed into an inverse BLR model to determine the optimal discrimination threshold of each gait feature. The statistical methodology is visualized in Fig. 1. Furthermore, mean AUC values were calculated for all gait parameters in each gait condition as well as for all gait conditions for each gait parameter. MATLAB® R2016b (The Mathworks Inc.) and Stata® 14.2 (Stata Corp.) software were used for data and statistical analyses. Results were considered significant at a *p*-value ≤ 0.05.

## Results

### Descriptive statistics

NPH patient characteristics are depicted in Table 1. The anthropometric measures between groups were comparable without statistically significant differences between the groups. 37 out of 55 (67.3%) patients suffered from all three symptoms of the Hakim’s triad. During gait assessment, “stops walking while talking” episodes were observed in 28 patients (50.9%), two patients (3.6%) showed a freezing of gait. No such phenomena were present in healthy subjects. NPH patients showed a reduced step length and walking speed with elongated double support phase, broadened base of support, and an increased variability of stride length (see Supplementary Table 1). In the course of disease, 51 patients (92.7%) received a CSF drainage procedure. Thereafter, gait improved in 43 (78.2%) patients, cognitive improvement was observed in 37 (67.3%) patients. 31 patients (56.4%) underwent VP shunt surgery.

**Table 1:** Sample characteristics of patients with idiopathic normal pressure hydrocephalus (NPH) and healthy subjects.

Sample characteristics	NPH	Healthy subjects	t-test
n (%)	55 (100)	55 (100)	
Age in years (SD)	72.6 (4.7)	70.5 (7.6)	n.s
Female (%)	18 (32.7)	27 (49.1)	n.s
Height in m (SD)	1.71 (0.09)	1.69 (0.25)	n.s
Leg length in m (SD)	0.88 (0.06)	0.91 (0.06)	n.s
Weight in kg (SD)	77.7 (13.4)	75.3 (16.2)	n.s
Disease duration in years^a^ (SD)	2.0 (1.5)	n.a	
Hakim’s triad^b^ (%)	37 (67.3)	n.a	
Gait disorder (%)	55 (100)	n.a	
Dementia (%)	48 (87.3)	n.a	
Urinary incontinence (%)	44 (80.0)	n.a	
Stops walking while talking^c^ (%)	28 (50.9)	0 (0)	
Freezing of gait^c^ (%)	2 (3.6)	0 (0)	
**Procedures in the course of disease**			
Lumbar drainage* (%)	51 (92.7)	n.a	
Single spinal tap test (%)	33 (60.0)	n.a	
Tuohy needle (%)	36 (65.5)	n.a	
VP shunt** (%)	31 (56.4)	n.a	

*NPH* idiopathic normal pressure hydrocephalus, *SD* standard deviation, *n.s*. not statistically significant, *n.a.* not applicable, *VP shunt* ventriculo-peritoneal shunt.

^a^Reported disease duration during first clinical visit due to NPH symptoms.

^b^Hakim’s triad, when patients present with all three symptoms: Gait disorder, dementia, and urinary incontinence.

^c^Presentation during clinical gait analysis, especially, when performing a dual task condition.

*Patients can receive a single spinal tap test, a lumbar drainage via Tuohy needle or both procedures in the course of disease.

**Implementation of a VP shunt in the course of disease.

### Gait parameter thresholds during preferred walking speed

**Figure 2:**
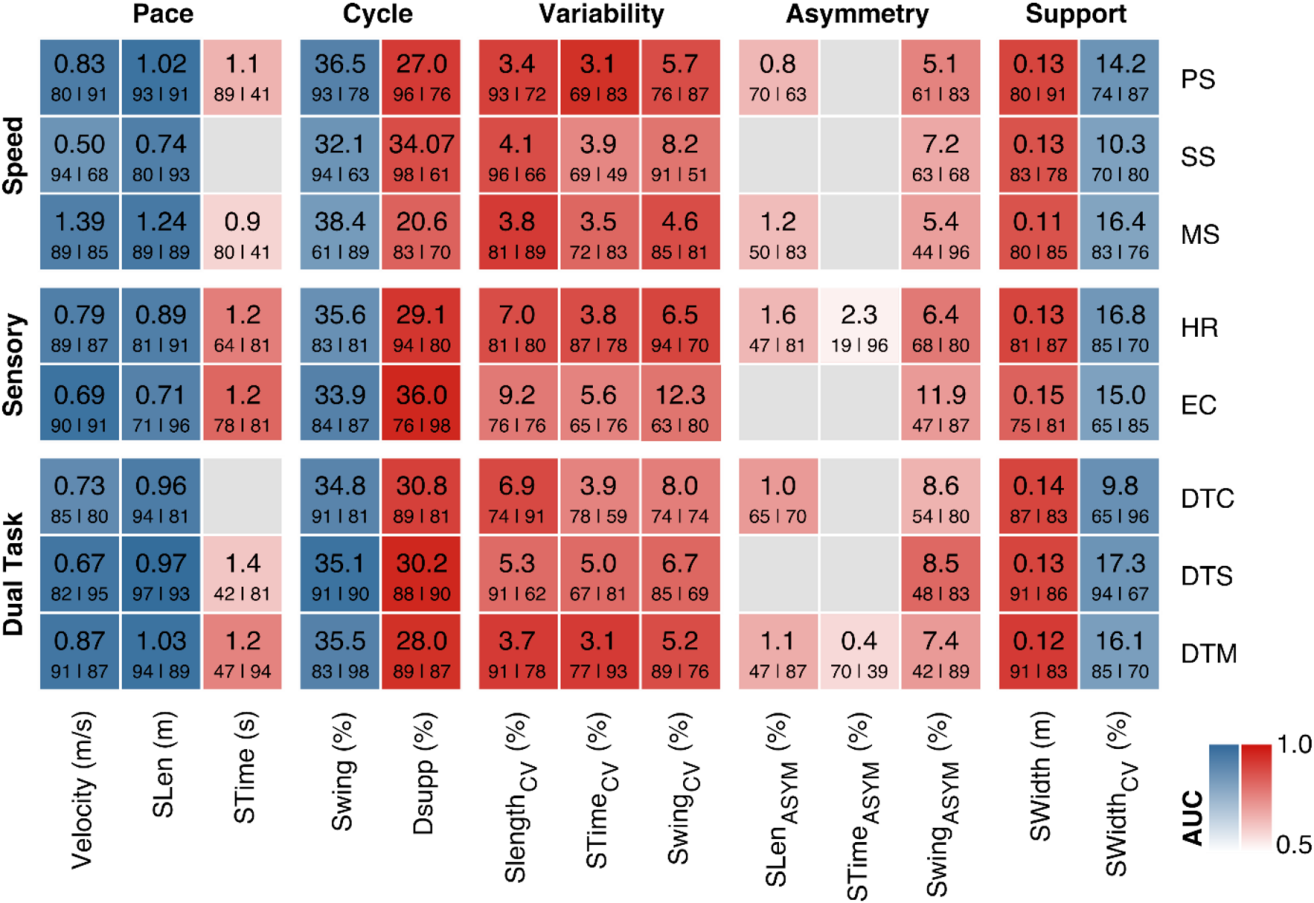
Overview of gait parameter thresholds, sensitivity and specificity values of patients with idiopathic normal pressure hydrocephalus (NPH) compared to age-matched healthy subjects. Gait parameters are arranged in five distinct global gait domains (x-axis). Different gait conditions are summarized in speed, sensory, and dual task conditions (y-axis). Each tile consists of the gait parameter threshold in its unit on the top, the small numbers on the bottom show the corresponding sensitivity (left) and specificity (right) in %. Tiles are color-coded according to the area under the curve (AUC) of the receiver operating characteristic (ROC) curve (see legend). Blue tiles illustrate that NPH patients fall below the threshold value, red tiles illustrate that NPH patients fall above the threshold value. Grey tiles indicate a non-significant regression fit. Abbreviations: Gait parameters: SLen: stride length; STime: stride time; Swing: percentage of swing phase; Dsupp: percentage of double support phase; CV: coefficient of variation; ASYM: asymmetry; SWidth: stride width. Gait conditions: PS: preferred walking speed; SS: slow walking speed; MS: maximal walking speed; HR: head reclination; EC: eyes closed; DTC: walking and serial 7 dual task; DTS: walking and verbal fluency dual task; DTM: walking and carrying a tray dual task.

The statistical approach is visualized in Fig. 1. AUC values of the classification models are presented in Table 2. Gait parameter thresholds, sensitivity as well as specificity values are illustrated for all gait conditions in Fig. 2.

**Table 2:** Overview of area under the curve (AUC) values of receiver operating characteristic (ROC) curves for gait parameters under different gait conditions for the classification models of idiopathic normal pressure hydrocephalus (NPH) patients and healthy subjects.

	Pace			Cycle		Variability			Asymmetry			Support		Mean of gait parameters
	Velocity (m/s)	SLen (m)	STime (s)	Swing (%)	Dsupp (%)	Slength_CV (%)	STime_CV (%)	Swing_CV (%)	SLen_ASYM (%)	STime_ASYM (%)	Swing_ASYM (%)	SWidth (m)	SWidth_CV (%)	
PS	0.93	**0.96**	0.66	0.91	0.91	0.9	0.84	0.87	0.65	0.53	0.76	0.89	0.85	0.82
SS	0.85	0.92	0.53	0.85	0.87	0.87	0.75	0.79	0.56	0.5	0.68	0.86	0.81	0.76
MS	0.92	0.94	0.59	0.83	0.83	0.91	0.82	0.87	0.64	0.51	0.7	0.86	0.8	0.79
HR	0.92	0.92	0.77	0.86	0.92	0.87	0.87	0.89	0.65	0.53	0.78	0.89	0.84	0.82
EC	**0.96**	0.91	0.82	0.91	**0.95**	0.78	0.76	0.79	0.5	0.5	0.71	0.83	0.81	0.79
DTC	0.88	0.93	0.63	0.9	0.9	0.88	0.76	0.77	0.71	0.5	0.66	0.89	0.86	0.79
DTS	**0.95**	**0.98**	0.62	**0.96**	**0.95**	0.81	0.81	0.82	0.57	0.53	0.68	0.89	0.85	0.80
DTM	**0.95**	**0.96**	0.76	0.91	0.93	0.91	0.91	0.87	0.67	0.58	0.71	0.91	0.82	**0.84**
Mean of gait conditions	0.92	**0.94**	0.67	0.89	0.91	0.87	0.82	0.83	0.62	0.52	0.71	0.88	0.83	

AUC values are highlighted in bold for values ≥ 0.95 and for the highest mean value. Abbreviations: Gait conditions: *PS* preferred walking speed; *SS* slow walking speed; *MS* maximal walking speed; *HR* head reclination; *EC* eyes closed; *DTC* walking and serial 7 dual task; *DTS* walking and verbal fluency dual task; *DTM* walking and carrying a tray dual task. Gait parameters: *SLen* stride length; *STime* stride time; *Swing* percentage of swing phase; *Dsupp* percentage of double support phase; *CV* coefficient of variation; *ASYM* asymmetry; *SWidth* stride width.

During preferred walking speed, the most distinct gait thresholds were variables in the Pace domain, namely stride length ≤ 1.02 m (sensitivity 0.93, specificity 0.91, AUC 0.96) and gait velocity ≤ 0.83 m/s (0.80, 0,91, AUC 0.93). Other significant variables include Cycle variables such as swing phase ≤ 36.5% (0.93, 0.78, AUC 0.91) and double support phase ≥ 27.0% (0.96, 0.76, AUC 0.91) as well as the Support variable stride width ≥ 0.13 m (0.80, 0.91, AUC 0.89). The most distinct variability parameter was stride length CV ≥ 3.4% (0.93, 0.72, AUC 0.90). Asymmetry parameters showed non-significant to only moderate discriminative power.

### Gait parameter thresholds during other gait conditions

The most noticeable thresholds were observed during DTS: Stride length ≤ 0.97 m (0.97, 0.93, AUC 0.98), swing phase ≤ 35.1% (0.91, 0.90, AUC 0.96), double support phase ≥ 30.2% (0.88, 0.90, AUC 0.95), and stride width ≥ 0.13 m (0.91, 0.86, AUC 0.89). During EC, the gait velocity threshold was ≤ 0.69 m/s (0.90, 0.91, AUC 0.96). The mean AUC values of all gait conditions for each gait parameter ranged from 0.52 (stride time asymmetry) to 0.94 (stride length). Further high overall AUC values were found for velocity (0.92) and for double support phase (0.91; see Table 2). Overall, the most distinct gait parameters thresholds were observed for the Pace, Cycle, and Support gait domains.

### AUC values and the influence of gait examination conditions

The mean AUC values of all examined gait parameters for each examination condition ranged from 0.76 (SS) to 0.84 (DTM). AUC values for PS and HR were 0.82. Highest single AUC values were found in DTS (stride length AUC: 0.98, swing phase AUC: 0.96, double support phase AUC: 0.95), DTM (stride length AUC: 0.96, velocity AUC: 0.95), EC (velocity AUC: 0.96, double support phase AUC: 0.95), and PS (stride length AUC: 0.96, velocity AUC: 0.93).

The most distinct dual task condition throughout all gait parameters was DTM with following noticeable threshold parameters: gait velocity ≤ 0.87 m/s (0.91, 0.87, AUC 0.95), stride length ≤ 1.03 m (0.94, 0.89, AUC 0.96), swing phase ≤ 35.5% (0.83, 0.98, AUC 0.91), double support phase ≥ 28.0% (0.89, 0.87, AUC 0.93), stride length CV ≥ 3.7% (0.91, 0.78, AUC 0.91), and stride width ≥ 0.12 m (0.91, 0.83, AUC 0.91).

## Discussion

The results of this study reveal that instrumented gait assessment is able to discriminate hypokinetic gait features of patients with NPH from walking performance of elderly healthy subjects with high accuracy. We found that the most discriminative gait parameters for NPH are present in gait features that are readily and easy to obtain by clinical gait assessment (e.g., step counts for a predefined walking distance, stopwatch). In addition, we observed that the variation of gait examination conditions affords a higher discriminatory power for single gait features. Most prominent examples were found for verbal fluency and motoric dual tasking.

### Gait features and their discriminatory characteristics

Outcomes of spatiotemporal gait parameters are consistent with previous studies of the hypokinetic gait disorder in NPH. Typical features are reduced stride lengths, a decreased walking speed, elongated double support phases, and a broadened base of support^9,11,23,24^. Parameters of the Pace gait domain (velocity, stride length) show the highest discriminatory power and best diagnostic values.

The estimated thresholds for spatiotemporal gait parameters lie mostly between previously reported gait parameter mean values of NPH patients and healthy subjects^11,23,25,26^. Some gait parameters of the healthy control group seem to differ slightly among several publication (e.g., lower gait velocity^25^, smaller stride length^26^), which might be due to varying baseline characteristics and/or a different gait assessment.

### Gait conditions and their discriminatory characteristics

Expanding the clinical gait assessment by additional examination conditions is useful for selected clinical settings, such as the early identification of patients with mild cognitive impairments. Previous studies emphasized the importance of examining dual task performance during evaluation of shunt candidacy in NPH during CSF tap test^16,27,28^. Other studies revealed that the evaluation of gait performance during single tasks is sufficient^15,29^. In line with this, the present results reveal a good to excellent classification accuracy across all examined conditions with highest mean AUC values for preferred walking and walking with a motoric dual tasking. In accordance to the latter finding, a comparative study between NPH and progressive supranuclear palsy (PSP; an atypical parkinsonian syndrome) patients revealed that motor dual task walking is the best condition to differentiate both diseases^17^. For Pace domain parameters such as velocity and stride length, our results reveal approximately equal to even higher values during dual task in healthy subjects.

Furthermore, single gait parameters show a very high discriminatory power and excellent sensitivity and specificity values in selected examination conditions. Most prominent is the evaluation of stride length during verbal fluency dual task walking. This finding might indicate that the utilization of these features might serve as a key diagnostic test to differentiate NPH from gait patterns of the healthy elderly population.

### Limitations

This study has several limitations. First, the reported gait parameter thresholds are established during a highly standardized walking examination procedure of steady-state walking. The thresholds cannot reflect parameters of walking in complex environmental situations or off-laboratory measurements. Therefore, it will in future be important to apply analogous ROC analysis procedures to gait data from other instrument-based sources (e.g. inertial sensors, wearables). Second, the NPH cohort consisted of patients with a mean age of 72.6 ± 4.7 years indicating that the calculated thresholds should be applied to patients in this age range. Due to the demographics of NPH with patients older than 80 years^5,30^, further studies are necessary to extend our knowledge regarding clinical meaningful gait thresholds for an older elderly population.

## Conclusions

In conclusion, the present study provides a comprehensive and systematic overview of clinical thresholds for spatial and temporal gait parameters that are useful for the diagnosis of NPH. We found that parameters of the pace domain during walking with self-selected speed and under dual task paradigms show the highest discriminatory power and excellent sensitivity and specificity for distinguishing the gait of patients with NPH from that of the healthy elderly walkers. By applying these analysis techniques to cohorts of clinical entities with similar gait patterns, future studies might support and promote the clinical status of instrumented gait assessment procedures for differential diagnosis.

## Supplementary Information

Supplementary Information.

## Data Availability

Anonymized datasets may be available upon reasonable request to qualified researchers within a reasonable time frame by contacting the corresponding, second, or last author.
