# Supplementary material for: Quantification of pathological gait parameter thresholds of idiopathic normal pressure hydrocephalus patients in clinical gait analysis
Source: Sci Rep. 2022 Oct 31;12:18295. doi: 10.1038/s41598-022-22692-1 (PMC9622747; doi:10.1038/s41598-022-22692-1)
Supplement: Supplementary file 1 — Supplementary Information. [file 41598_2022_22692_MOESM1_ESM.pdf]

Supplemental material

Supplementary table 1: Overview of spatiotemporal gait parameters during different gait conditions in idiopathic normal pressure hydrocephalus patients (NPH) and healthy subjects.

A) NPH | mean spatiotemporal gait parameters

	Velocity (m/s)	SLen (m)	STime (s)	Swing (%)	Dsupp (%)	Slength_CV (%)	STime_CV (%)	Swing_CV (%)	SLen_ASYM (%)	STime_ASYM (%)	Swing_ASYM (%)	SWidth (m)	SWidth_CV (%)
**PS**	0.67	0.80	1.2	32.9	34.2	7.3	4.0	8.4	1.1	0.7	6.8	0.16	12.5
**SS**	0.39	0.60	1.6	27.9	45.7	13.0	10.5	22.0	2.2	1.9	12.9	0.17	9.4
**MS**	1.10	1.02	0.9	37.2	25.7	6.4	4.3	6.7	1.3	1.1	5.3	0.15	13.6
**HR**	0.58	0.73	1.3	32.5	48.1	16.1	13.7	25.6	4.0	3.9	16.7	0.17	12.3
**EC**	0.52	0.64	1.3	30.7	45.8	15.8	12.8	24.8	3.2	2.8	16.6	0.18	14.4
**DTC**	0.55	0.70	1.3	29.8	44.6	11.6	12.9	16.9	1.9	1.5	11.0	0.18	10.2
**DTS**	0.54	0.69	1.4	29.8	41.5	11.0	9.8	15.1	1.2	1.3	9.9	0.18	10.9
**DTM**	0.68	0.80	1.2	32.9	38.0	7.8	7.5	13.5	1.1	1.7	8.9	0.16	12.4

B) NPH | standard deviations of spatiotemporal gait parameters

	Velocity (m/s)	SLen (m)	STime (s)	Swing (%)	Dsupp (%)	Slength_CV (%)	STime_CV (%)	Swing_CV (%)	SLen_ASYM (%)	STime_ASYM (%)	Swing_ASYM (%)	SWidth (m)	SWidth_CV (%)
**PS**	0.19	0.18	0.1	3.2	6.5	3.6	2.0	5.2	0.5	0.4	4.4	0.04	5.1
**SS**	0.10	0.16	0.3	4.0	8.4	17.8	14.9	34.9	6.3	5.9	16.0	0.05	4.7
**MS**	0.28	0.21	0.1	4.1	8.3	2.5	1.7	4.9	0.7	0.7	5.7	0.04	7.4
**HR**	0.19	0.20	0.3	7.1	34.1	19.9	23.0	41.4	11.8	12.8	26.9	0.04	7.1
**EC**	0.15	0.20	0.3	4.5	33.4	15.0	20.0	34.7	8.5	8.4	20.3	0.05	7.8
**DTC**	0.17	0.20	0.2	4.0	18.9	7.4	14.0	19.2	2.2	2.2	11.3	0.05	5.5
**DTS**	0.16	0.21	0.4	4.6	12.5	7.9	11.4	12.2	0.9	1.6	8.8	0.05	7.7
**DTM**	0.16	0.15	0.2	3.2	22.9	3.9	7.2	20.8	0.8	4.3	11.5	0.04	5.6

C) Healthy subjects | mean spatiotemporal gait parameters

	Velocity (m/s)	SLen (m)	STime (s)	Swing (%)	Dsupp (%)	Slength_CV (%)	STime_CV (%)	Swing_CV (%)	SLen_ASYM (%)	STime_ASYM (%)	Swing_ASYM (%)	SWidth (m)	SWidth_CV (%)
**PS**	1.06	1.19	1.1	38.1	23.9	3.0	2.4	4.3	0.9	0.8	3.5	0.10	21.4
**SS**	0.58	0.91	1.7	33.8	32.3	4.5	4.8	8.7	0.9	1.0	6.5	0.11	19.0
**MS**	1.61	1.44	0.9	40.6	18.6	2.7	2.8	4.1	0.9	1.0	3.1	0.10	23.4
**HR**	1.05	1.14	1.1	37.9	24.5	5.4	3.8	6.3	1.1	1.0	4.3	0.11	23.9
**EC**	0.95	1.01	1.1	37.0	26.4	7.6	4.6	9.3	1.5	1.0	7.2	0.12	25.9
**DTC**	0.94	1.11	1.3	37.1	26.3	4.1	4.9	8.3	0.8	1.1	6.1	0.11	20.1
**DTS**	0.99	1.17	1.2	38.4	23.6	5.5	4.0	6.9	1.3	1.0	5.3	0.10	23.1
**DTM**	1.11	1.18	1.1	38.2	23.8	2.9	2.4	4.9	0.7	0.6	4.0	0.10	20.1

D) Healthy subjects | standard deviations of spatiotemporal gait parameters

	Velocity (m/s)	SLen (m)	STime (s)	Swing (%)	Dsupp (%)	Slength_CV (%)	STime_CV (%)	Swing_CV (%)	SLen_ASYM (%)	STime_ASYM (%)	Swing_ASYM (%)	SWidth (m)	SWidth_CV (%)
**PS**	0.19	0.14	0.1	2.4	4.6	1.4	1.3	1.4	0.6	0.9	2.0	0.03	8.9
**SS**	0.17	0.14	0.3	4.3	8.5	2.4	2.0	3.3	0.6	0.9	4.2	0.04	18.2
**MS**	0.26	0.18	0.1	2.0	3.9	1.3	1.9	3.2	0.5	0.5	2.6	0.03	11.4
**HR**	0.25	0.19	0.1	3.1	6.3	5.2	4.2	3.4	0.6	1.1	3.2	0.03	10.5
**EC**	0.19	0.18	0.1	2.3	4.7	4.4	2.5	4.5	0.9	0.7	4.7	0.04	11.4
**DTC**	0.27	0.19	0.3	3.7	7.8	2.5	4.8	7.1	0.6	1.2	5.1	0.04	11.3
**DTS**	0.26	0.19	0.2	2.9	5.8	4.2	3.4	4.6	0.7	0.9	4.6	0.03	12.6
**DTM**	0.19	0.14	0.1	2.2	4.4	1.3	1.9	4.0	0.4	0.3	3.5	0.03	7.9

Abbreviations: Gait parameters: SLen: stride length; STime: stride time; Swing: percentage of swing phase; Dsupp: percentage of double support phase; CV: coefficient of variation; ASYM: asymmetry; SWidth: step width. | Gait conditions: PS: preferred walking speed; SS: slow walking speed; MS: maximal walking speed; HR: head reclination; EC: eyes closed; DTC: walking and serial 7 dual task; DTS: walking and verbal fluency dual task; DTM: walking and carrying a tray dual task.

Supplementary table 2: Overview of mean number of strides in different gait conditions in idiopathic normal pressure hydrocephalus patients (NPH) and healthy subjects.

Gait condition	Number of strides (SD)	
	NPH	Healthy subjects
**PS**	41.2 (19.8)	23.2 (14.7)
**SS**	54.9 (27.8)	33.7 (23.0)
**MS**	32.0 (15.4)	19.0 (11.9)
**HR**	45.1 (21.0)	22.3 (8.0)
**EC**	47.8 (20.6)	24.8 (7.8)
**DTC**	42.8 (18.5)	22.8 (8.0)
**DTS**	50.8 (25.6)	22.2 (9.3)
**DTM**	37.1 (13.5)	20.4 (5.9)

Abbreviations: SD: standard deviation; Gait conditions: PS: preferred walking speed; SS: slow walking speed; MS: maximal walking speed; HR: head reclination; EC: eyes closed; DTC: walking and serial 7 dual task; DTS: walking and verbal fluency dual task; DTM: walking and carrying a tray dual task.
